# *NOD* promoter-controlled *AtIRT1* expression functions synergistically with *NAS* and *FERRITIN* genes to increase iron in rice grains

**DOI:** 10.1007/s11103-015-0404-0

**Published:** 2015-11-11

**Authors:** Kulaporn Boonyaves, Wilhelm Gruissem, Navreet K. Bhullar

**Affiliations:** Plant Biotechnology, Department of Biology, ETH Zurich (Swiss Federal Institute of Technology Zurich), Universitaetsstrasse 2, 8092 Zurich, Switzerland

**Keywords:** Rice, Iron biofortification, Iron-regulated metal transporter, Rice endosperm

## Abstract

Rice is a staple food for over half of the world’s population, but it contains only low amounts of bioavailable micronutrients for human nutrition. Consequently, micronutrient deficiency is a widespread health problem among people who depend primarily on rice as their staple food. Iron deficiency anemia is one of the most serious forms of malnutrition. Biofortification of rice grains for increased iron content is an effective strategy to reduce iron deficiency. Unlike other grass species, rice takes up iron as Fe(II) via the IRON REGULATED TRANSPORTER (IRT) in addition to Fe(III)-phytosiderophore chelates. We expressed Arabidopsis *IRT1* (*AtIRT1*) under control of the *Medicago sativaEARLY NODULIN 12B* promoter in our previously developed high-iron NFP rice lines expressing *NICOTIANAMINE SYNTHASE* (*AtNAS1*) and *FERRITIN*. Transgenic rice lines expressing *AtIRT1* alone had significant increases in iron and combined with *NAS* and *FERRITIN* increased iron to 9.6 µg/g DW in the polished grains that is 2.2-fold higher as compared to NFP lines. The grains of *AtIRT1* lines also accumulated more copper and zinc but not manganese. Our results demonstrate that the concerted expression of *AtIRT1*, *AtNAS1* and *PvFERRITIN* synergistically increases iron in both polished and unpolished rice grains. *AtIRT1* is therefore a valuable transporter for iron biofortification programs when used in combination with other genes encoding iron transporters and/or storage proteins.

**Electronic supplementary material:**

The online version of this article (doi:10.1007/s11103-015-0404-0) contains supplementary material, which is available to authorized users.

## Introduction

Iron deficiency is the most common and widespread nutrient deficiency, particularly among children as well as women of childbearing age. Considering anemia as an indicator, 43 % of children, 38 % of pregnant woman and 29 % of non-pregnant women worldwide are estimated to be iron deficient (Steven et al. 2013). The main causes of iron deficiency include low bioavailable iron in the diet, increased requirements in pregnancy, or excessive blood loss caused by injuries, intestinal parasitic infection or menstruation. Increased dietary diversification, iron supplementation or food fortification are recommended interventions to overcome iron deficiency. Food fortification is considered the most effective and safest intervention in malaria-affected regions where iron supplementation leads to increased severity of the infectious disease (Oppenheimer et al. 1986; Sazawal et al. 2006). However, iron fortification of food is problematic since bioavailable iron compounds often react with other food components to cause off-flavors, color changes, or fat oxidation (Abbaspour et al. 2014). Agronomic practices, conventional breeding and/or genetic engineering are alternative approaches to enhance the nutritional content of staple crops. Rice is the main staple food for more than half of the world’s population and therefore an important target crop for biofortification. Rice is a poor source of dietary iron because the micronutrient-rich aleurone, bran and husk of the grain are removed during polishing. Most of the cultivated mega-rice varieties contain only around 2 µg iron per gram of grain after polishing (Bouis et al. 2011). Improving iron content in the rice endosperm using conventional breeding approaches alone has not been successful because the endosperm iron content has a very narrow range in the rice germplasm, as is the case for most other micronutrients (Slamet-Loedin et al. 2015). Therefore, genetic engineering is an important option for increasing iron content in rice endosperm (polished grains).

Genetic engineering approaches for iron biofortification reported to date have focused on overexpression of genes encoding the iron storage protein FERRITIN, iron transporters, and/or increasing production of iron chelating compounds. Endosperm-specific expression of FERRITIN, which can store up to 4500 Fe molecules in its central cavity, have increased iron content 2- to 3.7-fold in polished rice grains (Goto et al. 1999; Lucca et al. 2001; Oliva et al. 2014; Qu et al. 2005; Vasconcelos et al. 2003). Most graminaceous plants including rice utilize a chelation-based strategy for iron uptake and release phytosiderophores (PS) into the soil that bind ferric iron (Takagi 1976; Takagi et al. 1984). The PS-Fe(III) complexes are then transported into the root cells by specific transporters. All PS of the mugeneic acid (MA) family are synthesized from S-adenosyl-l-methionine via a conserved pathway of nicotianamine synthase (NAS), nicotianamine aminotransferase (NAAT) and deoxymugineic acid synthase (DMAS) (Kobayashi and Nishizawa 2012). Overexpression of the *NAS* gene resulted in a two- to four-fold increase in rice endosperm iron content (Masuda et al. 2009; Johnson et al. 2011; Lee et al. 2012). Other MA biosynthesis-related genes, for example, barley *IRON DEFICIENCY*-*SPECIFIC 2* (*IDS2*), *3* (*IDS3*) and *NAAT* have also been introduced into rice, either alone or in combination with *NAS* (Kobayashi et al. 2008; Masuda et al. 2008; Mori et al. 2001; Suzuki et al. 2008; Takahashi et al. 2001) as well as with other iron transporters. Elevated iron levels were reported in transgenic rice lines overexpressing the combination of *FERRITIN*, *NAS* and *RICE YELLOW STRIPE*-*LIKE 2* (*OsYSL2*) or of *FERRITIN* and *NAS* together with the barley *NAAT* and *IDS3* genes (Aung et al. 2013; Masuda et al. 2012, 2013b). Several of the YELLOW STRIPE PROTEIN (YS) and YS-LIKE PROTEIN (YSL) family transporters have been evaluated in iron biofortification strategies. Transgenic expression of barley *YS1* in rice increased leaf iron content by 1.5-fold, but no increase in grain iron was reported (Gomez-Galera et al. 2012). Constitutive expression of *OsYSL15* resulted in a 1.2-fold increase of iron content in rice grains (Lee et al. 2009) and a phloem-specific overexpression of *OsYSL2* increased iron content fourfold in rice endosperm (Ishimaru et al. 2010). Enhanced iron translocation from flag leaves to rice grains by reducing the expression of the vacuolar iron transport related genes *OsVIT1* or *OsVIT2* also resulted in a 1.8-fold increase of endosperm iron content (Bashir et al. 2013; Zhang et al. 2012). Additionally, constitutive overexpression of *OsIRO2,* which encodes an iron deficiency-inducible basic helix-loop-helix (bHLH) transcription factor regulating key genes involved in iron homeostasis increased iron content of the unpolished grains threefold when grown on calcareous soil (Ogo et al. 2011). The endosperm-specific expression of *FERRITIN* and *PHYTASE* combined with constitutive expression of *NAS* (NFP rice) gave a sixfold increase in the endosperm iron content in polished grains (Wirth et al. 2009). However, most of the iron biofortification strategies reported to date have not yet reached the target of 15 µg Fe/g (dry weight) in the rice endosperm (Bouis et al. 2011), except for the expression of a single *OsNAS2* gene that produced lines with 14 and 19 µg/g iron in the polished grains (Johnson et al. 2011).

The IRON REGULATED METAL TRANSPORTER (IRT) is a member of the ZINC-REGULATED TRANSPORTER/IRON-REGULATED TRANSPORTER (ZRT/IRT1)-RELATED PROTEIN (ZIP) transporter family (Eng et al. 1998) and candidate for iron biofortification. In non-graminaceous plants, IRT facilitates Fe(II) uptake from the rhizosphere to the root cells (Eide et al. 1996; Guerinot 2000). Arabidopsis *IRT1* is predominantly expressed in roots and upregulated in iron deficiency conditions (Eide et al. 1996). Unlike other graminaceous monocots that utilize chelation based strategy for Fe(III) uptake, rice can directly take up Fe(II) in water-logged paddy fields abundant in Fe(II) (Cheng et al. 2007). *OsIRT1* and *OsIRT2* encode Fe(II) transporters that are induced in iron deficient conditions (Ishimaru et al. 2006), confirming that rice has a functional Fe(II) uptake mechanism. We have introduced the Arabidopsis *IRT1* gene into high-iron NFP rice lines (Wirth et al. 2009) under the control of *pMsENOD12B*, a *Medicago sativa* early nodulin gene promoter that is active in rice vascular tissue and root epidermal cells (Terada et al. 2001; Werthmüller 2000). NFP rice contains around 6 µg/gDW iron in polished grains when grown hydroponically and around 4 µg/g DW when grown in soil, in contrast to Taipei 309 control plants whose iron content in polished grains ranges between 1 and 2 µg/g DW. Expression of *AtIRT1* in NFP lines (Wirth et al. 2009) significantly increases iron content in polished grains (9.6 µg/g DW) of soil grown plants, making *AtIRT1* a strong candidate for iron biofortification of rice.

## Results

### *MsENOD12B*-controlled expression of *AtIRT1* increases iron content in rice grains

**Fig. 1:**
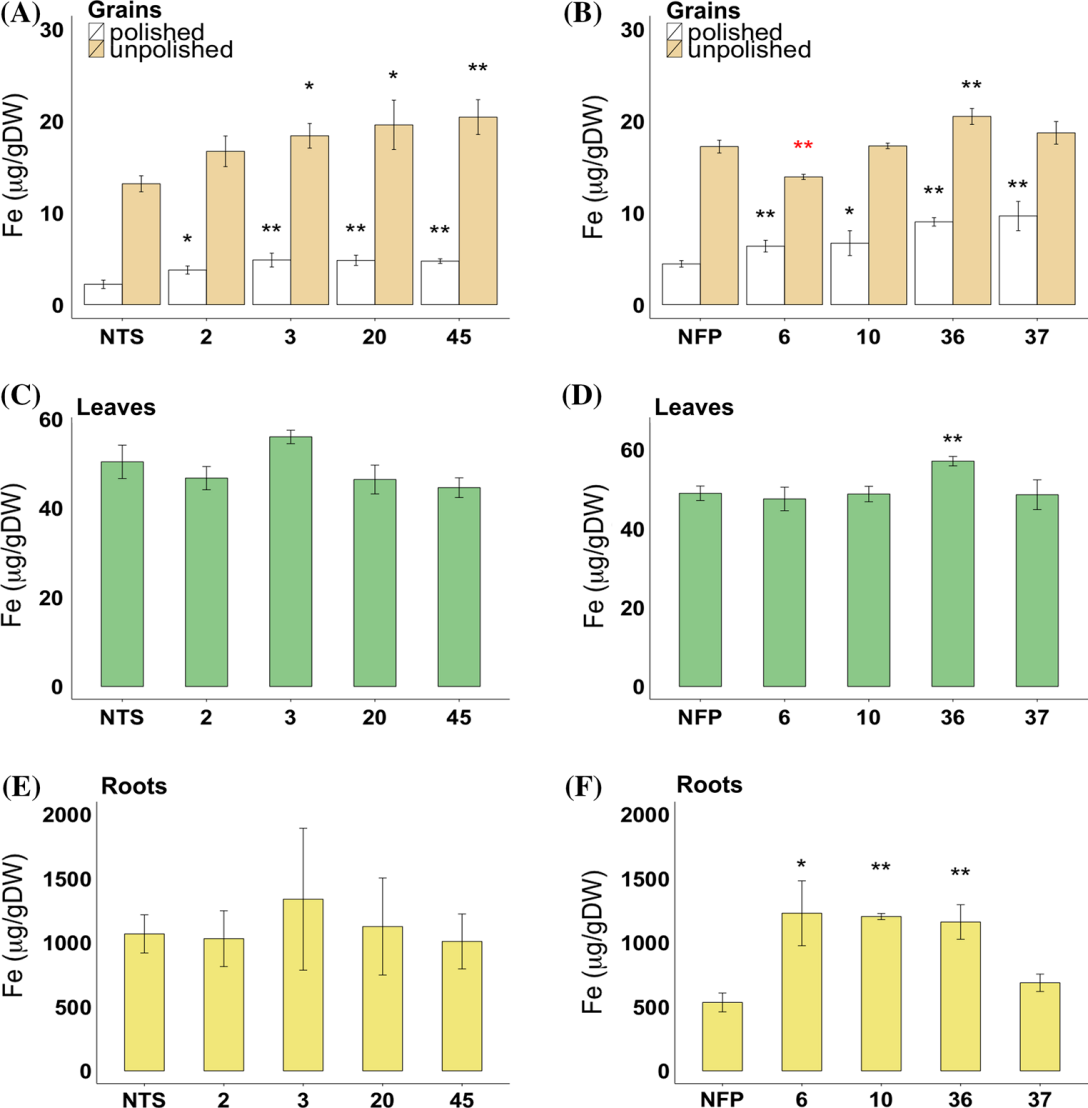
Iron content in the grains, leaves and roots of *AtIRT1* expressing lines. Iron content in the T_3_ polished and unpolished grains, IRT-TP309 (**a**) and IRT-NFP (**b**). Iron content in the leaves of IRT-TP309 (**c**) and IRT-NFP (**d**) plants. *Numbers* indicate the selected lines. Iron content in the roots of IRT-TP309 (**e**) and IRT-NFP (**f**) plants. *Values* are the average of three biological replicates (±SD). *Black* and *red* *asterisks* above the *bars* indicate statistically higher and lower significant values calculated using Student’s *T* test, respectively, in comparison to the NTS or NFP controls (*P < 0.05; **P < 0.01). *NTS* non-transgenic sibling control

*AtIRT1* under the control of *MsENOD12B* promoter was transformed into Taipei 309 (TP309) and NFP rice (Wirth et al. 2009). Nine and twelve transgenic lines containing single transgene insertions in the TP309 background (IRT-TP309) and NFP background (IRT-NFP), respectively, were selected for growing the T_1_ generation. Based on the iron content in T_2_ polished grains (Supplementary Fig. 1), four IRT-TP309 (2, 3, 20 and 45) and IRT-NFP lines (6, 10, 36 and 37) each were selected for further analysis and were grown into T_2_ generation plants. T_3_ grains of IRT-TP309 and IRT-NFP lines had increased iron content in comparison to their non-transgenic controls (Fig. 1).

The iron content in polished grains of IRT-TP309 lines ranged from 3.78 to 4.86 µg/g DW as compared to 2.28 µg/g DW in non-transgenic sibling (NTS) control lines (Fig. 1a). Unpolished grains of IRT-TP309 lines had increased iron content ranging from 16.72 to 20.45 µg/g DW compared to 14.01 µg/g DW in NTS grains. The endosperm iron in line 3 was the highest with a 2.1-fold increase (4.86 µg/g) as compared to the control plants. Similarly, the IRT-NFP lines had up to 2.2-fold higher iron content (ranging from 6.38 to 9.67 µg/g DW) in polished grains as compared to the NFP lines (Fig. 1b). Unpolished grains of transgenic IRT-NFP lines contained 13.93 to 20.53 µg/g DW iron, which is variable as compared to the NFP control. These increase in iron content demonstrate the utility of the *pMsENOD12B::AtIRT1* construct for iron biofortification in rice.

**Fig. 2:**
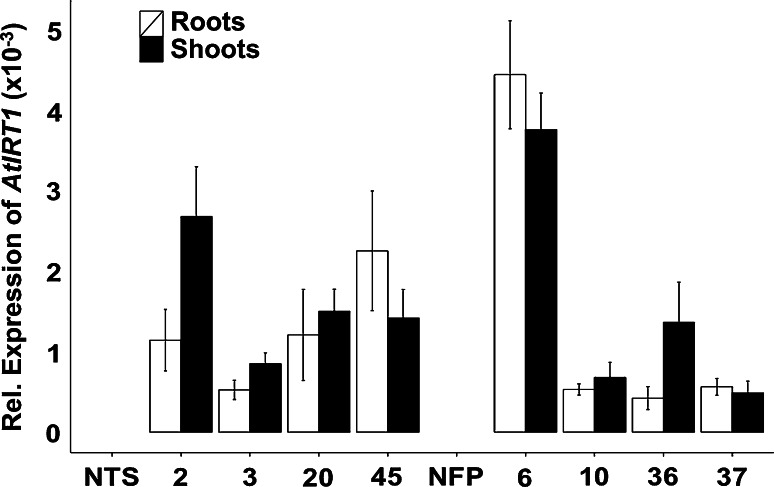
Relative expression of transgene *AtIRT1* in the independent transgenic lines. Relative *AtIRT1* expression in root (*white bar*) and shoot (*black bar*) samples of 5-day-old seedlings in T_3_ generation is estimated. No expression of *AtIRT1* was observed in NTS and NFP controls. The data were normalized with the endogenous expression of *Os01g0147200* and *Os11g0661400*. *Values* are the average of three biological replicates (±SD)

Leaf iron content of IRT-TP309 and IRT-NFP lines did not show significant differences to control plants, except IRT-NFP line 36 that had 1.2-fold higher leaf iron content (Fig. 1c, d). No significant change was observed in root iron content of IRT-TP309 lines, whereas three IRT-NFP lines had up to 2.3-fold increased iron as compared to NFP roots (Fig. 1e, f). However, the root iron content of these three IRT-NFP lines was not significantly different from TP309 (1068.12 µg/g DW iron) because of the reduced root iron content of the NFP siblings (Supplementary Fig. 2). Preliminary phenotypic characterization of T_2_ generation transgenic plants grown in soil showed variability for various scored parameters (days to flowering, plant height, SPAD value, one thousand grain weight; Supplementary Table 1). All transgenic lines had moderate but variable expression levels of *AtIRT1* in both shoots and roots (Fig. 2), indicating that activity of the *MsENOD12B* promoter is not entirely restricted to rice vascular tissue and root epidermal cells as previously reported (Terada et al. 2001; Werthmüller 2000). IRT-TP309 line 45 had the highest *AtIRT1* expression in the root and line 2 in the shoot, while IRT-NFP line 6 had high *AtIRT1* expression levels in both shoot and root compared to the other IRT-NFP lines. The *AtIRT1* expression differences among the independent transgenic lines did not correlate with the increased iron content observed in the grains, leaves and roots of these plants. Additionally, expression of several endogenous genes related to iron homeostasis was examined in selected transgenic lines. Although there were notable differences in the expression of the tested endogenous genes among the independent transformed lines, most genes showed a consistent expression (Supplementary Fig. 3; Supplementary Fig. 4).

### Grains of *AtIRT1* lines accumulate higher copper and zinc but not manganese

**Fig. 3:**
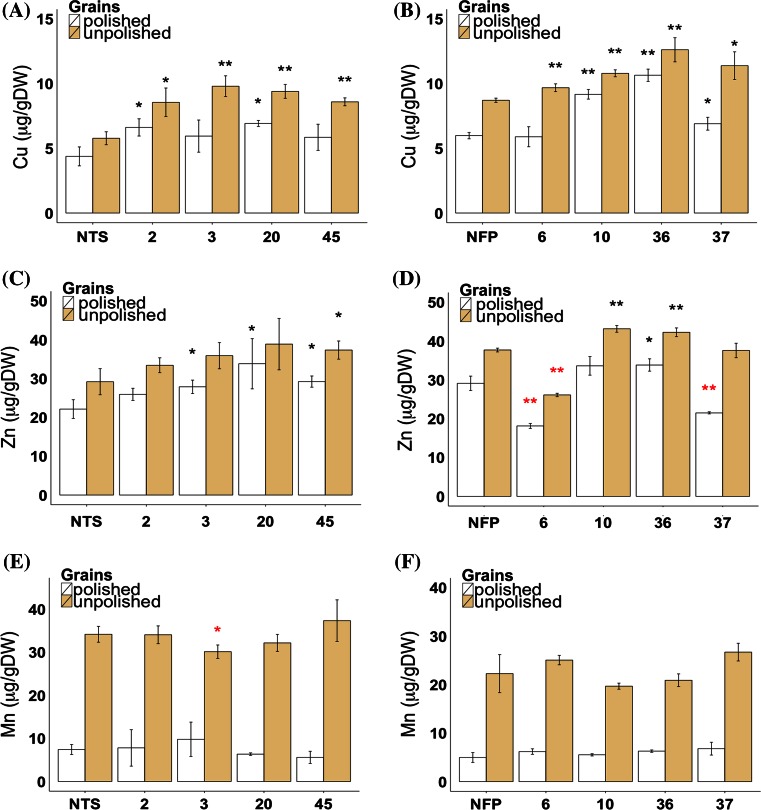
Copper, zinc and manganese concentration in the grains of *AtIRT1* expressing transgenic lines. T_3_ grains of the transgenic IRT-TP309 and IRT-NFP lines were analyzed for copper (**a**, **b**), zinc (**c**, **d**) and manganese content (**e**, **f**). *Numbers* indicate the selected lines. *Values* are the average of three biological replicates (±SD). *Black* and *red* *asterisks* above the *bars* indicate statistically higher and lower significant values calculated using Student’s *T* test, respectively, in comparison to the NTS or NFP controls (*P < 0.05; **P < 0.01)

Among the metal ions copper, manganese and zinc were investigated in the T_3_ grains of *AtIRT1* expressing lines. Copper was significantly increased in unpolished grains of both IRT-TP309 and IRT-NFP lines as compared to control lines (Fig. 3a, b). In the IRT-TP309 lines, the unpolished grains contained 8.56–9.80 µg/g DW copper (1.7-fold). However, only IRT-TP309 lines 2 and 20 showed a significant copper increase in polished grains with the highest copper content at 6.92 µg/g DW. Similarly, all four IRT-NFP lines had significant increases of copper in unpolished grains (up to 1.4-fold) while lines 10, 36 and 37 also had up to 1.8-fold copper increase in polished grains as compared to the NFP control (Fig. 3b). Furthermore, zinc content in polished grains was increased up to 1.5-fold in IRT-TP309 lines 3, 20 and 45 (Fig. 3c) and IRT-NFP line 36 (Fig. 3d) while IRT-NFP lines 6 and 37 showed decreased grain zinc content as compared to NFP plants (Fig. 3d). Similar increases of zinc content in the unpolished grains were observed for IRT-TP309 line 45 (1.3-fold) and IRT-NFP lines 10 and 36 (Fig. 3c, d). Unlike zinc and copper, the manganese content remained unchanged in grains of all the *AtIRT1* expressing lines, except in IRT-TP309 line 3 in which manganese was slightly decreased (Fig. 3e, f).

## Discussion

Iron biofortification of rice endosperm to dietary sufficient RDA levels has proven much more challenging compared to previously successful β-carotene biofortification of rice (Paine et al. 2005). This is likely due to the complex network of genes controlling iron homeostasis, including uptake, transport and storage, as well as iron transporters that can transport a broader range of metals (Thomine and Vert 2013). Efforts have been continuing for more than a decade to enhance iron levels in polished rice grains. Breeding for higher endosperm iron content is not possible because of the low endosperm iron variation in rice germplasm (Meng et al. 2005). Rice has therefore been transformed with genes encoding transporters mediating iron uptake and/or translocation, iron storage proteins and/or transcription factors controlling iron deficiency responses (Masuda et al. 2013a). Until recently, most transgenic iron biofortification approaches focused on phytosiderophore (PS) synthesis-related genes and the iron storage protein FERRITIN. Overexpression of *NAS* was a preferred approach because in addition to an increased production of PS in transgenic plants synthesis of nicotianamine (NA), a metal-chelator for internal iron transport (Stephan et al. 1996; Stephan and Scholz 1993), was also increased. Concerted expression of various gene combinations i.e., expressing genes related to uptake, translocation and storage from a single construct have generally been more successful than single transgene strategies. However, the transgene combinations might not always have a synergistic effect. Some of the studies also attempted increasing the sink in the rice endosperm by raising the *FERRITIN* expression levels, but with not much success. For example, overexpression of *FERRITIN* using both endosperm-specific *GLOBULIN* and *GLUTELIN* promoters did not increase iron content compared to expression of a single *FERRITIN* gene (Qu et al. 2005), most likely because of limiting iron mobilization from leaves during grain filling. Polished rice grains obtained from T_2_ transgenic lines overexpressing six transgenes (*FERRITIN* under the control of *GLOBULIN* and *GLUTELIN* promoters, barley *NAAT*-*A*, *NAAT*-*B*, *NAS1* and *IDS3*), did not lead to further increases in iron content compared to dual *FERRITIN* overexpressing lines (Masuda et al. 2013b). Although different transgenic strategies have met varied levels of success to increase the iron content in both polished and unpolished rice grains, novel strategies are required to achieve further iron increases (at least 15 µg/g DW in polished grains) to meet the recommended dietary allowance levels.

The iron deficiency induced expression of *OsIRT1* and *OsIRT2*, which are homologs of *IRT1* in rice, encouraged the use of *IRT* in rice iron biofortification strategies (Lee and An 2009; Tan et al. 2015; Xiong et al. 2014). Transgenic rice expressing *OsIRT1* under the control of the maize *UBIQUITIN* promoter were tolerant to iron deficiency and had over 1.1-fold increased iron content in the shoot and mature seeds but showed an aberrant growth phenotype in paddy fields (Lee and An 2009). The peanut *IRT1* expressed under the control of an iron deficiency-inducible promoter in rice resulted in improved tolerance to low Fe availability in calcareous soils (Xiong et al. 2014). More recently, *CaMV35S* promoter-driven expression of apple *IRT1* in rice resulted in increased iron content (up to 30.5 mg/kg) in mature seeds (Tan et al. 2015), but data on iron content in polished grains is not available from any of these studies. Thus, it is possible that a novel strategy of expressing an Fe(II) transporter such as IRT1 together with NAS and FERRITIN can further increase iron in the rice endosperm. To establish a proof of concept, we transformed the Arabidopsis *IRT1* gene into our previously developed high-iron NFP rice (Wirth et al. 2009) in which *NAS* and *FERRITIN* genes synergistically increase grain iron content. To restrict the expression of *AtIRT1* in transgenic rice we used the promoter of the early nodulin gene *ENOD12B* from alfalfa (*Medicago sativa*), which is expressed in nodules and also at low levels in roots, flowers, stems, and leaves (Bauer et al. 1994). Transgenic rice plants expressing a *pMsENOD12B::GUS* construct showed that the promoter is active in the vascular tissue as well as in epidermis and root hair cells (Terada et al. 2001; Werthmüller 2000). Our transgenic rice lines expressing *pMsENOD12B::AtIRT1* in combination with *NAS* and *FERRITIN* genes had higher iron content in polished and unpolished grains as compared to the NFP plants. Together, our results show that restricted expression of *AtIRT1* acts synergistically with *NAS* and *FERRITIN* to effectively increase iron in polished rice grains.

IRT1 has a high affinity for Fe(II) (Rogers et al. 2000) but also transports other divalent metal ions such as manganese, cadmium and zinc. Most of our *AtIRT1* transgenic plants had increased grain copper content and in some lines, increased zinc content but no differences in manganese. However, these changes in the metal ion concentrations may not be a direct effect of *AtIRT1* expression and the role of other metal homeostasis-related genes cannot be excluded. *AtIRT1* expression may modulate the expression of other metal homeostasis related genes (Antosiewicz et al. 2014; Wang et al. 2013). We previously showed that several metal transport-related genes, including *OsIRT1*, *OsZIP1*, *OsZIP3*, *OsZIP4*, and *OsYSL6* were upregulated in NFP lines (Wang et al. 2013). *OsNAS3* and *OsDMAS1,* which encode enzymes in MA biosynthesis, were also upregulated in NFP rice when grown under low iron conditions (Wang et al. 2013). MA and NA also have a significant role in the transport of essential metals such as copper, iron, manganese and zinc (Haydon and Cobbett 2007). Therefore, similar modulation of metal homeostasis related endogenous genes could promote the uptake of other metal ions in addition to iron in the *AtIRT1* transgenic rice lines. These possibilities can now be investigated in more detail using the IRT-TP309 and IRT-NFP lines. Together, restricted expression of *AtIRT1* is an effective strategy for iron biofortification when used in combination with other iron transporters and/or iron storage proteins. In order to achieve further increases in iron, the biofortification strategies need to consider approaches going beyond the uptake of iron by the roots. It has become evident that even if increased amounts of iron can be transported into the plant, it does not necessarily reach the grain. Therefore, future strategies need to explore candidate genes that can promote effective translocation within the plant in combination with transporters that can increase availability of free iron for transport and storage into the grains.

## Materials and methods

### DNA construct, rice transformation and plant growth conditions

The construct expressing the Arabidopsis *IRT1* gene [GenBank:U27590] under the control of *MsENOD12B* promoter was kindly provided by Dr. Christof Sautter (ETH Zurich). It was generated by amplifying *AtIRT1* from a cDNA obtained from Arabidopsis roots using forward primer GGATCCTCTCATGAAAACAATCTT and reverse primer TCTAGAGCAGCAAAAGTTTTATTTATTT. The fragment was cut with *Bam*HI and *Xba*I before inserting it into pMSB containing the *MsENOD12B* promoter (Terada et al. 2001). The transformation vector was transferred via *Agrobacterium tumefaciens* strain EHA105 (Hood et al. 1993) to *Oryza sativa* ssp. *japonica* cv. Taipei 309 (TP309) and to the previously developed high iron transgenic NFP rice (Wirth et al. 2009). Transformation, selection on hygromycin and regeneration were conducted as described by Nishimura et al. (2006). Plants were grown in a plant growth chamber with 60 % humidity/28 °C/16 h light and 8 h dark. Genomic DNA extraction was performed on leaves of 2-week-old plants using CTAB solution as described by Vasudevan et al. (2014). The presence of the transgene was verified by PCR using the primers *AtIRT1*fw, 5′-TGATGCTACCTTGAAGCTTAG-3′ and *AtIRT1*rv, 5′-TCAACTGCGCCGGAAGAATG-3′. Southern blot hybridization using digoxigenin (DIG) labeling was performed on *Bam*HI digested genomic DNA of the transgenic lines to select for transformants with a single copy transgene insertion. The *AtIRT1* fragment (889 base pairs) amplified by the *AtIRT1* specific primers was used as a probe to detect the transgene. Example of Southern hybridization analysis is shown in Supplementary Fig. 5. The selected transformants, NFP and TP309 plants were grown in commercial soil (Klasmann-Deilmann GmbH, Germany) in the greenhouse conditions with 80 % humidity/30 °C/12 h light and 60 % humidity/22 °C/12 h dark. Mature grains were collected at 5 weeks after flowering for metal quantification. For metal quantification in the vegetative parts, shoot and root of 1-month-old seedlings of T_3_ generation were collected.

### Metal ion measurements

Grain samples were de-husked to obtained unpolished brown grains. In order to obtain polished grains, the de-husked grains were processed with a grain polisher (Kett grain polisher ‘Pearlest’, Kett Electric Laboratory, Tokyo, Japan) for 1 min. Shoot and root samples were dried at 60 °C for 5 days. The samples were ground and 200 mg of each ground sample was boiled in 15 ml of 65 % v/v HNO_3_ solution at 120 °C for 90 min. Three ml of 30 % v/v H_2_O_2_ were subsequently added and continuously boiled at 120 °C for 90 min. Metal concentrations were determined using inductively coupled plasma-optical emission spectroscopy (ICP-OES) (Varian Vista-MPX CCD Simultaneous ICP-OES). The wavelength used for iron, zinc, manganese and copper were 238.204, 213.857, 257.610, and 324.754, respectively. The National Institute of Standards and Technology (NIST) rice flour standard 1658a was treated and analyzed in the same manner and used as quality control for every measurement. Data were analyzed using Student’s *t* test. The criteria of alpha = 0.05 and alpha = 0.01 was used to determine statistically significant differences among the tested lines.

### Quantitative real-time PCR

Total RNA was extracted from root and shoot of 5-day-old seedlings in T_3_ generation using Trizol^®^ reagent (Invitrogen, USA) and the RNA was treated with DNase I (Thermo Fisher Scientific Inc., USA) to remove genomic DNA contamination. First-strand cDNA was synthesized by RevertAid™ first strand cDNA synthesis kit (Thermo Fisher Scientific Inc., USA). Quantitative real-time PCR (qRT-PCR) was performed using Taqman hydrolysis probes (Roche, Switzerland) on 7500 FAST Real Time PCR system (Applied Biosystem, Inc., USA). Total reaction volume of 25 µl included 0.5 µl cDNA, 2.25 µl forward primer, 2.25 µl reverse primer, 0.25 µl probe (Roche Ltd., Switzerland), 12.5 µl Taqman^®^ Gene Expression Mastermix (Applied Biosystems Ltd., USA) and 7.25 µl H_2_O. Primers were designed using Roche primer design website (https://lifescience.roche.com/shop/CategoryDisplay?catalogId=10001&tab=&identifier=Universal+Probe+Library&langId=-1&storeId=15006). Probe number and primer sequences are provided in Supplementary Table 2. The Ct value was obtained from 7500 Fast System Software (Applied Biosystems, Inc., USA). Primer efficiency was calculated by LinReg PCR (Tuomi et al. 2010). The data of qRT-PCR was normalized as described by Schefe et al. (2006).

## Electronic supplementary material

Supplementary material 1 (PDF 5318 kb)
