# Supplementary material for: NOD promoter-controlled AtIRT1 expression functions synergistically with NAS and FERRITIN genes to increase iron in rice grains
Source: Plant Mol Biol. 2015 Nov 11;90:207–15. doi: 10.1007/s11103-015-0404-0 (PMC4717176; doi:10.1007/s11103-015-0404-0)
Supplement: Supplementary file 1 — Supplementary material 1 (PDF 5318 kb) [file 11103_2015_404_MOESM1_ESM.pdf]

***NOD* promoter-controlled *AtIRT1* expression functions synergistically with *NAS* and *FERRITIN* genes to increase iron in rice grains**

Plant Molecular Biology

Kulaporn Boonyaves[1], Wilhelm Gruissem[1] and Navreet K. Bhullar[1,*]

[1]Plant Biotechnology, Department of Biology, ETH Zurich (Swiss Federal Institute of Technology Zurich), Universitaetsstrasse 2, 8092 Zurich, Switzerland

[*]Correspondence:
Dr. Navreet Bhullar
Plant biotechnology
LFW E17
ETH Zurich
Universitaetsstrasse 2,
8092 Zurich, Switzerland
e-mail: bhullarn@ethz.ch

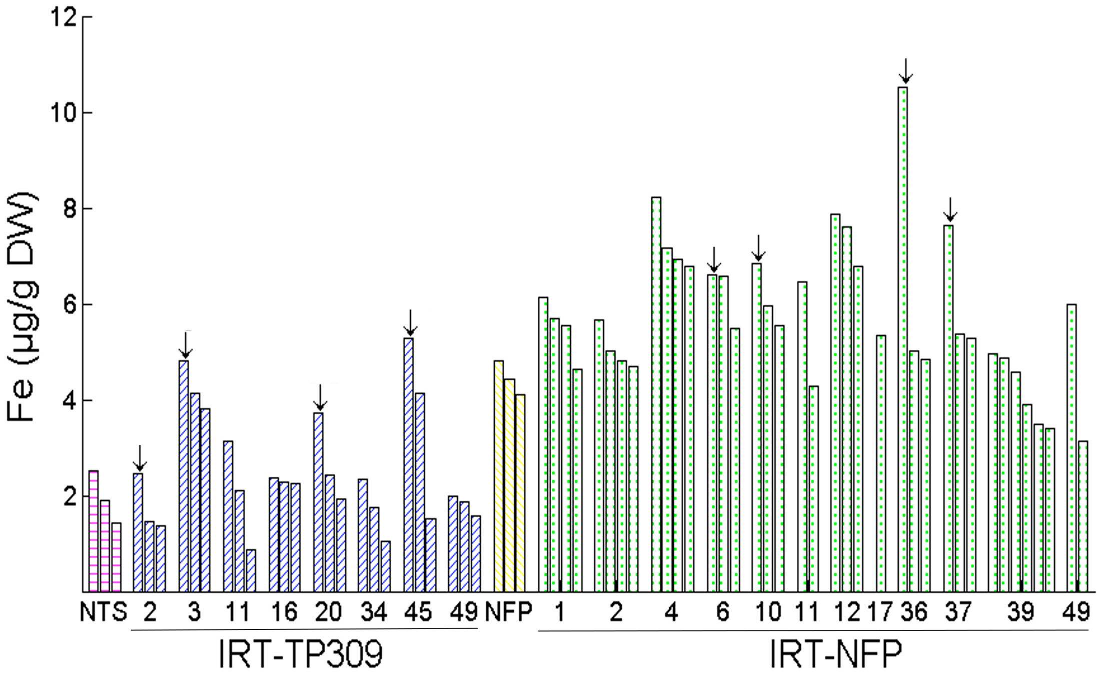

Supplementary Fig. 1 Iron concentration in polished T_2 grains of IRT-TP309 and IRT-NFP lines. Iron content in polished grains of IRT-TP309 lines and IRT-NFP lines are compared with NTS and NFP control. The bars for each line represent individual plants. Arrows indicate selected plants from each line that were grown further in T_2 generation.

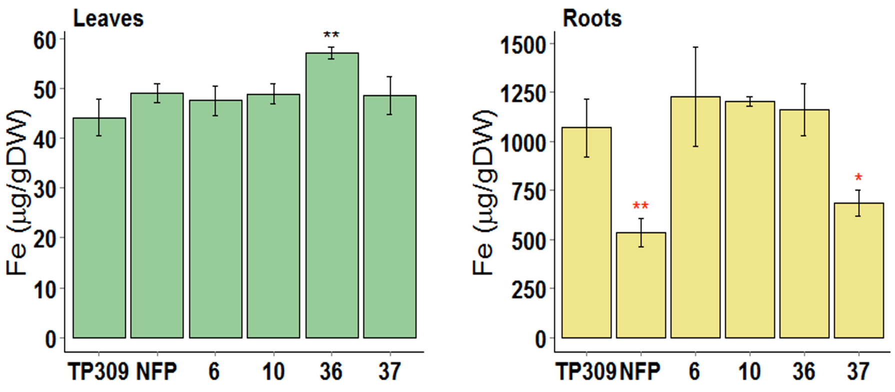

Supplementary Fig. 2 Iron content in leaves and roots of IRT-NFP lines in comparison to TP309. The bars for each line represent three individual plants. Values are the average of three biological replicates (± standard deviation). Black and red asterisks above the bars indicated statistically higher and lower significant values calculated using Student's T-Test, respectively, in comparison to the TP309 (*, $P < 0.05$, **, $P < 0.01$).

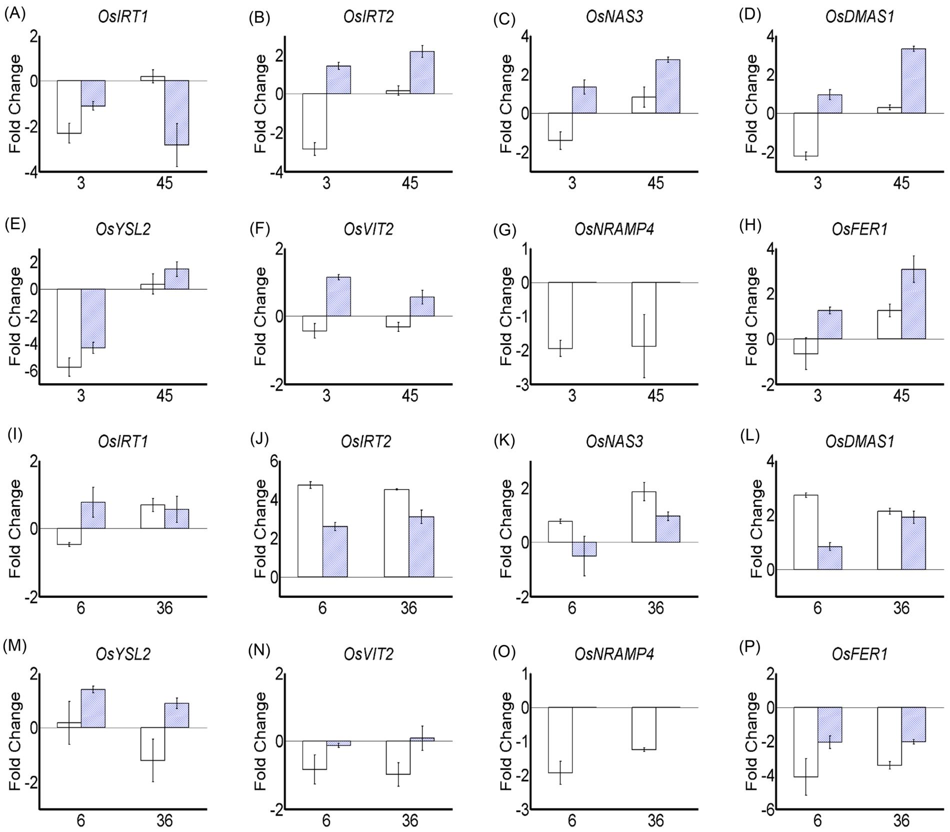

Supplementary Fig. 3 Expression patterns of endogenous genes related to metal homeostasis in the *AtIRT1* transgenic lines. Fold change of relative expression (log_2) of endogenous genes encoding metal homeostasis related proteins *IRON REGULATED METAL TRANSPORTER 1* and *2* (*OsIRT1* and *OsIRT2*), *NICOTIANAMINE SYNTHASE 3* (*OsNAS3*), *DEOXYMUGINEIC ACID SYNTHASE 1* (*OsDMAS1*), *YELLOW STRIPE-LIKE TRANSPORTER 2* (*OsYSL2*), *VACUOLAR IRON TRANSPORTER 2* (*OsVIT2*), *NATURAL RESISTANCE ASSOCIATED MACROPHAGE PROTEIN 4* (*OsNRAMP4*), and *FERRITIN 1* (*OsFER1*) is presented. The NTS and NFP plants were used as controls for IRT-TP309 and IRT-NFP lines, respectively. Expression was measured in roots (white bar) and shoots (blue bar) of 5-day-old T_3 seedlings. Values are the average of three biological replicates (± standard deviation). The data were normalized with the endogenous expression of *Os01g0147200* and

Os11g0661400. Relative expression levels of NTS and NFP control plants are shown in Supplementary Fig. 4

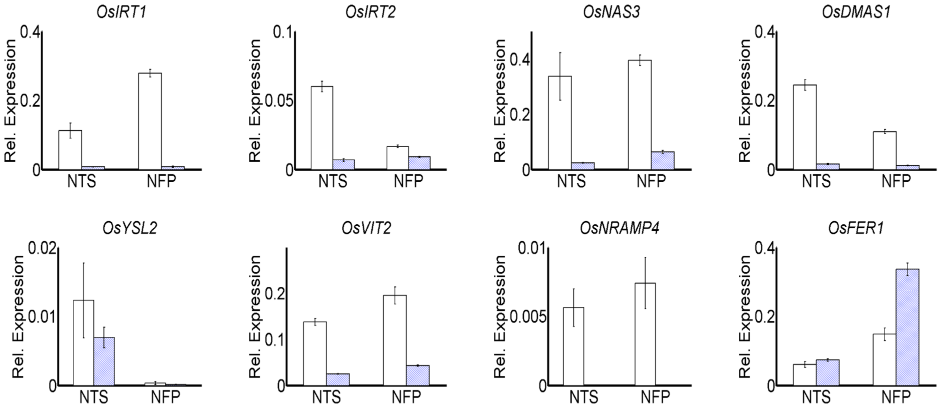

Supplementary Fig. 4 Relative expression of endogenous metal homeostasis related genes (*OsIRT1, OsIRT2, OsNAS3, OsDMAS1, OsYSL2, OsVIT2, OsNRAMP4,* and *OsFER1*) in roots (white bar) and shoots (blue bar) of NTS and NFP control plants. The data were normalized with the endogenous expression of *Os01g0147200* and *Os11g0661400*.

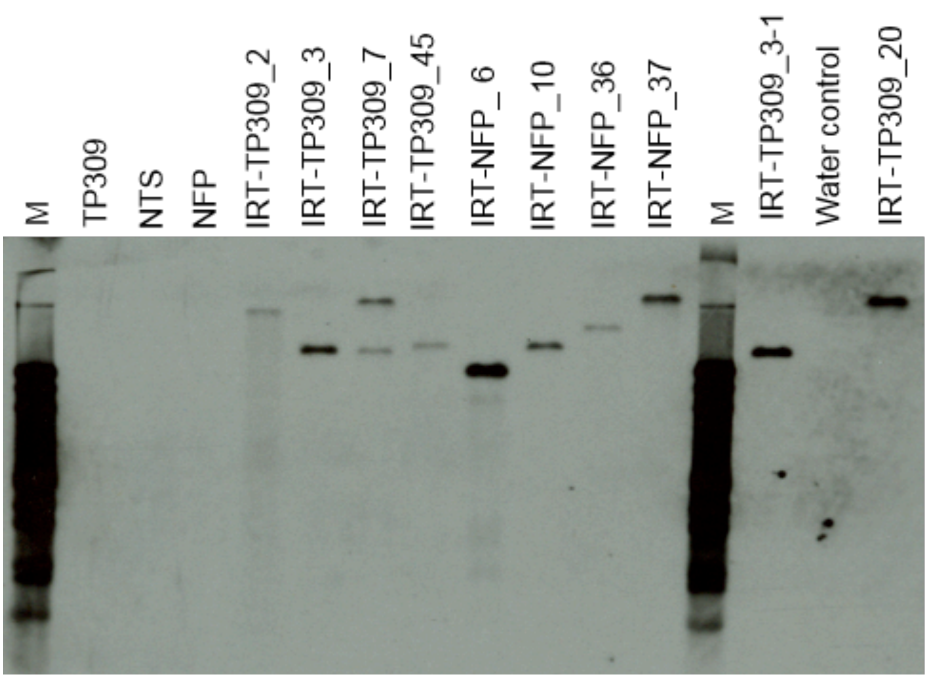

Supplementary Fig. 5 Example of Southern hybridization analysis in IRT-TP309 and IRT-NFP transgenic lines. Genomic DNA was digested by *Bam*HI. The transgenic IRT-TP309 line 2, 3, 20, and 45 and the transgenic IRT-NFP line 6, 10, 36 and 37 were detected to contain single copy of transgene insertion and were chosen for further analysis. M: DIG labeled DNA molecular weight marker; TP309: Taipei 309; NTS: non-transgenic sibling; NFP: the transgenic rice line containing *AtNAS1*, *PvFERRITIN* and *AfPHYTASE* insertion in the TP309 background; IRT-TP309: transgenic lines containing *AtIRT1* insertion in the TP309 background; IRT-NFP: transgenic lines containing *AtIRT1* insertion in the NFP background.

Supplementary Table 1 Phenotypic greenhouse performance of transgenic *AtIRT1* rice lines. The parameters including days to flowering, plant height, SPAD value, and 1000 grain weight (1000GW) were recorded. Values are the average of three biological replicates (± standard deviation). Transgenic IRT-TP309 and IRT-NFP plants were compared to NTS and TP309, respectively. Black and red asterisks indicated statistically higher and lower significant values calculated using Student's T-Test, respectively, in comparison to the NTS or TP309 controls (*, $P < 0.05$, **, $P < 0.01$).

	**Plant line**	**Days to flowering**	**Height (cm)**	**SPAD value**	**1000GW (g)**
**NTS**		88.7 ± 4.6	78.8 ± 1.0	43.6 ± 0.4	20.5 ± 0.8
**IRT-TP309**	2	92.3 ± 4.0	70.9 ± 0.7 **	44.0 ± 2.3	21.0 ± 0.1
	3	92.3 ± 4.0	80.5 ± 4.6	44.1 ± 5.2	25.8 ± 1.6 **
	20	100 ± 3.6 *	52.8 ± 5.2 **	47.9 ± 0.7 **	22.6 ± 0.8 *
	45	118 ± 7.1 **	62.0 ± 4.5 **	49.1 ± 1.0 **	22.7 ± 0.5 *
**TP309**		88.7 ± 2.3	85.0 ± 0.5	42.8 ± 1.5	23.7 ± 0.6
**NFP**		74.0 ± 0.0**	82.5 ± 3.2	46.0 ± 1.6	26.2 ± 0.9*
**IRT-NFP**	6	79.7 ± 3.5*	78.6 ± 2.3 **	45.5 ± 2.1	25.9 ± 1.9
	10	87.3 ± 4.5	66.3± 13.3	51.3 ± 5.2	25.2 ± 2.1
	36	95.0 ± 0.0 **	59.2 ± 2.9 **	43.8 ± 1.8	22.5 ± 1.7
	37	118.0 ± 8.7 **	60.3 ± 4.7 **	46.9 ± 0.8*	22.9 ± 1.7

Supplementary Table 2 List of primers and probes used for quantitative gene expression analysis (qRT-PCR)

Nr.	Gene	Accession ID	Forward primer	Reverse primer	TaqMan probe
**1**	*AtIRT1*	NM_118089	tcgaaggcatgggtcttg	acgccataacaaatttcttcatatt	65
**2**	*OsIRT1*	AB070226	gacactggtgcccattctg	gaggatggggatggagga	63
**3**	*OsIRT2*	AB126086	tcaggaatcgcgtcattgt	agcccgatcaccactgag	105
**4**	*OsNAS3*	AB023819	gaggaggaggtgatcgagaa	atcaccagctccgtgaaca	70
**5**	*OsDMAS1*	AB269906	aaaagctcgacaccctgct	tccctcagcttcctctgct	39
**6**	*OsYSL2*	AB164646	tggagcttcttccagtggtt	gaggctgaaatcaaaatagaacg	22
**7**	*OsVIT2*	Os09g0396900	ggcctcggagggtatctg	acagtatgtccgcgatctcc	15
**8**	*OsNRAMP4*	AK102180	gccattggcttcctagatcc	aagatgacccacagaagctca	78
**9**	*OsFER1*	AF519570	aggggatgccttgtatgct	cggtcagctgtggatcatt	148
**10**	*Os01g0147200*	NM_001048546	agcagctgaaagcaccaaa	cacgcccttcaacactgag	124
**11**	*Os11g0661400*	NM_001074942	tcgctataccacccctcttg	acgatggaggacgaaggtag	7
